# Molecular Characterization of *TP53* Gene in Human Populations Exposed to Low-Dose Ionizing Radiation

**DOI:** 10.1155/2013/303486

**Published:** 2013-03-17

**Authors:** Igor Brasil-Costa, Dayse O. Alencar, Milene Raiol-Moraes, Igor A. Pessoa, Alexandre W. M. Brito, Schneyder R. Jati, Sidney E. B. Santos, Rommel M. R. Burbano, Ândrea K. C. Ribeiro-dos-Santos

**Affiliations:** ^1^Laboratory of Human and Medical Genetics, Biological Sciences Institute, Federal University of Pará (UFPA), Augusto Correa Street, Number 01, CEP 66075-110 Belém, PA, Brazil; ^2^Epstein-Barr Virus Laboratory, Virology Section, Evandro Chagas Institute, BR-316 Highway Km 7, CEP 67030-000 Ananindeua, PA, Brazil; ^3^Human Cytogenetics Laboratory, Biological Sciences Institute, Federal University of Pará (UFPA), Augusto Correa Street, Number 01, CEP 66075-110 Belém, PA, Brazil

## Abstract

Ionizing radiation, such as that emitted by uranium, may cause mutations and consequently lead to neoplasia in human cells. The *TP53* gene acts to maintain genomic integrity and constitutes an important biomarker of susceptibility. The present study investigated the main alterations observed in exons 4, 5, 6, 7, and 8 of the *TP53* gene and adjacent introns in Amazonian populations exposed to radioactivity. Samples were collected from 163 individuals. Occurrence of the following alterations was observed: (i) a missense exchange in exon 4 (Arg72Pro); (ii) 2 synonymous exchanges, 1 in exon 5 (His179His), and another in exon 6 (Arg213Arg); (iii) 4 intronic exchanges, 3 in intron 7 (C → T at position 13.436; C → T at position 13.491; T → G at position 13.511) and 1 in intron 8 (T → G at position 13.958). Alteration of codon 72 was found to be an important risk factor for cancer development (*P* = 0.024; OR = 6.48; CI: 1.29–32.64) when adjusted for age and smoking. Thus, *TP53* gene may be an important biomarker for carcinogenesis susceptibility in human populations exposed to ionizing radiation.

## 1. Introduction

Exposure to genotoxic chemical and physical agents may result in genetic damage in human populations. These agents may interfere with normal cell development, disrupt normal cell growth and proliferation control, and lead to an increased risk of neoplasia development [1, 2]. 

 Ionizing radiation is an example of a physical genotoxic agent that may induce direct (via energy absorption) or indirect (via high production of reactive free radicals) damage to DNA molecules. The impact of radioactive energy on DNA may result in the destruction of bases and hydrogen bridges and the breakage of single- or double-stranded DNA [3, 4].

Indirectly, this type of radiation may provoke mutations due to the high production of reactive free radicals formed by the breakup of molecules through ionization. These radicals react with any surrounding molecules, producing a new, structurally different molecule. When a nucleotide is affected, may occur a mismatch and consequent change after DNA replication [3]. 

 The identification and use of biomarkers to monitor populations under excessive and/or continuous exposure to toxic and radioactive agents are important. Biomarkers may be used to assess probability of disease and to determine any possible increase in the risk of developing long-term health problems [5, 6].

### 1.1. The Element Uranium

 Uranium is a heavy, malleable, weakly paramagnetic metal that has radioactive properties. Uranium has several isotopes, the most important of which are radioactive and are presented in nature with masses of 238, 235, and 234 [7, 8]. 

 Servomaa and Rytomaa [9, 10] have reported both *in vitro* and *in vivo* cell transformations caused by uranium. Miller et al. [11] first demonstrated the induction of malignant transformation of human cells exposed to uranium compounds. In complex organisms, it is difficult to predict the consequences of uranium exposure; however, it is important to monitor populations that have been exposed to the element [12–14].

**Figure 1:**
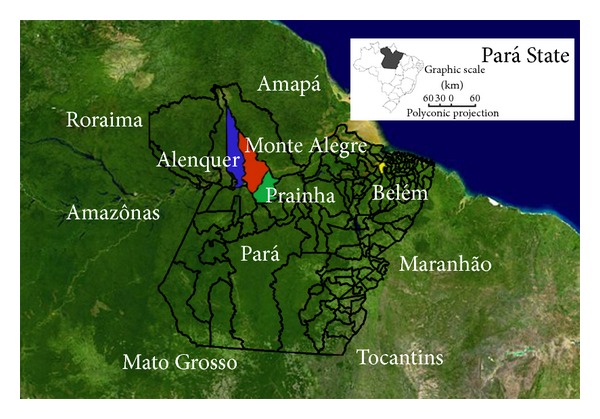
Map of the study region. Red: Monte Alegre municipality; blue: Alenquer municipality; green: Prainha municipality; yellow: Belém city ([17, 18], adapted).

According to official records, Brazil has one of the largest reserves of uranium in the world, at approximately 309,000 tons of U_2_O_8_, a figure that may be an underestimation because only a quarter of the Brazilian territory has been properly explored. According to the Companhia de Recursos Minerais—CPRM (Mineral Resources Company)—the municipality of Monte Alegre in the northeast of Pará State (Amazon) is one of the largest mineralization areas of uranium in the world, with uranium present in over 800 km^2^ extending into the municipalities of Prainha and Alenquer (Figure 1). Furthermore, most homes in Monte Alegre have been built with rocks taken from areas with uranium reserves [15].

Among the decay products of uranium, the most important is ^222^Rn, which is responsible for a large part of the radioactivity in the atmosphere. The concentrations of ^222^Rn inside the houses built in the Monte Alegre region ranged from 88 ± 80 to 338 ± 19 Bq m^−3^. In other areas of the Amazon, the concentration of ^222^Rn is much lower (28 ± 3 Bq m^−3^) [19].

 Ponnaiya et al. [20] determined that radioactivity causes genomic instability and chromosomal breakage in human fibroblasts. This study also reported no significant relationship between radiation dose and genomic instability, which may occur even at low radiation doses. Biological variables that make some individuals more sensitive to the effects of ionizing radiation than others may also exist.

A broader approach to better understand the biological effects of ionizing radiation on humans should include measuring the amount of radiation exposure and analyzing the genetic constitutions of exposed individuals. Good biomarkers for genetic susceptibility would typically be involved in cell cycle control, apoptosis, DNA repair, and the metabolism of genotoxic agents [21, 22]. In this study, *TP53*, an important tumor suppressor gene and susceptibility marker, was selected for investigation. 

### 1.2. Tumor Protein p53 Gene (*TP53*)

 The gene *TP53 *(17p13.1) encodes a 53-kD protein (p53) [23, 24]. Mutations in this gene may produce genomic instability because p53 controls numerous cell processes, including the detection of and response to DNA damage and the response to oncogenic activation signals [22, 25]. *TP53 *dysfunction may result in deregulation of these processes, changes in the cellular response to radiation, and alterations in radiosensitivity [26].

The most frequent mutations of *TP53 *occur between residues 102 and 296 in the region that controls DNA binding. This region includes “mutation hotspots” located between exons 5 and 8 that have a higher percentage of mutations, comprising approximately 30% of all known mutations [27, 28].

 In addition to these “mutation hotspots,” exons 5, 6, 7, and 8 contain approximately 94% of the characterized *TP53 *mutations and therefore constitute a region of interest for screening [29]. *TP53* also has several single-nucleotide polymorphisms (SNPs). Most of these SNPs are located within introns, outside the conserved splice regions. There are two main SNPs that modify amino acids: (i) a T → C change at the first nucleotide of codon 47 (rs1800371) that results in an amino acid change from serine to proline; and (ii) a G → C change at the second nucleotide of codon 72 (rs1042522) that modifies arginine to proline (Arg72Pro) [28]. 

 The most frequent and most studied amino acid exchange in *TP53* is that of codon 72. This exchange is important for susceptibility to neoplasia development and has different prevalences among distinct ethnic groups [30–32].

 Other types of polymorphisms include insertions or deletions of segments, such as the duplication of 16 base pairs (5′ ACCTGGAGGGCTGGGG 3′) in the third intron, known as PIN3 (rs17878362) [33]. This duplication may increase the risk of developing breast cancer [34], colorectal cancer [35], and lung cancer [36], although the associations are still controversial [37, 38].

 Given the inaccuracy of the stipulated safety limits of radioactivity and the biological importance of *TP53* in the susceptibility to genetic diseases, this study sought to identify *TP53* mutations in populations exposed to radioactivity.

## 2. Methods

### 2.1. Research Subjects

 The study enlisted 163 individuals who were approached at public health care centers in eastern Pará State municipalities that keep radioactivity records. Due to difficulties in accessing the study area, volunteers were invited to participate in the study through announcements made on the local radio station. The subjects were randomly selected, were not biologically related, and had lived on site for over a year. Sixty-four individuals were from the municipality of Monte Alegre, 50 from the municipality of Prainha, and 49 from the municipality of Alenquer.

 This study was submitted to the Ethics Committee of Research with Human Beings of the Center of Tropical Medicine under protocol number 002/2007 and was approved in accordance with Brazilian Health Council/Health Ministry Resolution 196/96. All participating individuals signed a free informed consent form and answered a social, clinical, and environmental survey for population profiling and testing of possible factors for risk of mutagenesis and/or carcinogenesis [39–41]; profiles included data on age, gender, ethnic origin, occupation, smoking history, drinking habits, personal history of cancer, family history of cancer, history of miscarriage, and reproductive difficulty. Additionally, 222 healthy individuals from Belém (another Amazon population not exposed to uranium) were selected as controls (Figure 1).

### 2.2. Genetic Analysis

 Approximately 5 mL of peripheral blood was collected and mixed with the anticoagulant EDTA. DNA was extracted with phenol chloroform and precipitated with ethanol, as described by Sambrook et al. [42]. Polymerase chain reaction (PCR) was used to amplify four regions of *TP53 *with high mutation frequencies.

The primers for each investigated region were developed with the specialized software programs Primer3 [43] and Fast PCR [44] (Table 1). PCR was performed using concentrations of 0.1 *μ*M of deoxynucleotide triphosphate, 1.5 *μ*M of MgCl_2_, 0.1 *μ*M of each of the primers, 10 mM of Tris-HCl, pH = 8.3, 50 mM of KCl, 1 U of *Taq *polymerase, and 100 ng of sample DNA.

PCR was performed at 95°C/4 min, followed by 35 cycles of 95°C/1 min, 60°C/1 min, and 72°C/1 min, with a final extension of 72°C/60 min. PCR conditions were similar for all amplified regions, with the exception of region 4, for which the annealing temperature was 58°C. The region in which the PIN3 duplication occurs was also amplified, and the size of the amplified fragment was determined. The DNA fragments were separated using an ABI PRISM 3130 Genetic Analyzer (Life Technologies, CA, USA) and were analyzed with the program GeneMapper v3.2 (Life Technologies, CA, USA).

The amplified regions were submitted to direct sequencing by the chain-termination method [45] using a Big Dye Terminator kit (v.3.1), which uses the enzyme AmpliTaq DNA (Life Technologies, CA, USA). The primers were the same as those used in the PCR step. The sequencing reaction was performed in a 20 *μ*L volume containing 15 *μ*L of water, 1 *μ*L of amplified PCR product, 3.5 *μ*L of Big Dye Terminator, and 0.5 *μ*L of each primer. The samples were then separated by capillary electrophoresis in a 3130 Genetic Analyzer for sequencing (Life Technologies, CA, USA) using the fragment migration polymer POP-7. The sequencing results were obtained from electropherograms in FASTA format, visualized with the software ChromasPro version 1.33 [46], and analyzed for the presence of nucleotide polymorphisms in relation to the normal reference sequences from the genetic database GenBank [16].

### 2.3. Statistical Analysis

Allelic frequencies were estimated by gene counting. For each sample from the investigated populations, the estimated allele frequences were fitted to the expected Hardy-Weinberg equilibrium values with a chi-square test (*χ* ^2^) using the statistics software BioEstat 5.0 [47]. Estimates of linkage disequilibrium were performed using the statistical package Arlequin 3.0 [48]. The populations were compared for social, clinical, and environmental factors, and the frequencies of changes were found by *χ* ^2^ test, residue analysis, Fisher's exact test, Kruskal-Wallis, and simple and multiple logistic regression using the software BioEstat 5.0. *P* < 0.05 was used for statistical association and a confidence interval of 95% for logistic regression.

The difference between allele frequencies found in the present investigation and those described for the general [16] and control (Belém) populations was evaluated with the statistics software CLUMP version 11 [49].

## 3. Results

### 3.1. Sampling Description

**Figure 2:**
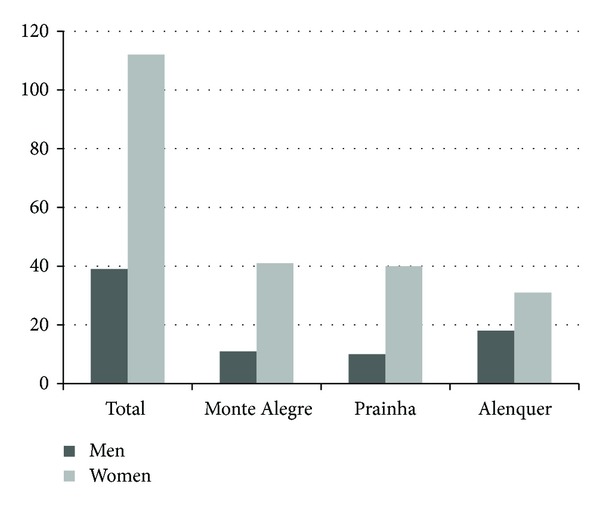
Gender distribution in the study population.

 Of the 163 samples originally obtained, 151 were used for genetic analysis: 52 from Monte Alegre, 50 from Prainha, and 49 from Alenquer. Comparisons of the three populations by gender using residue analysis indicated that the Alenquer population was different from the others (*P* < 0.05), with a relatively larger number of males (Figure 2).

**Figure 3:**
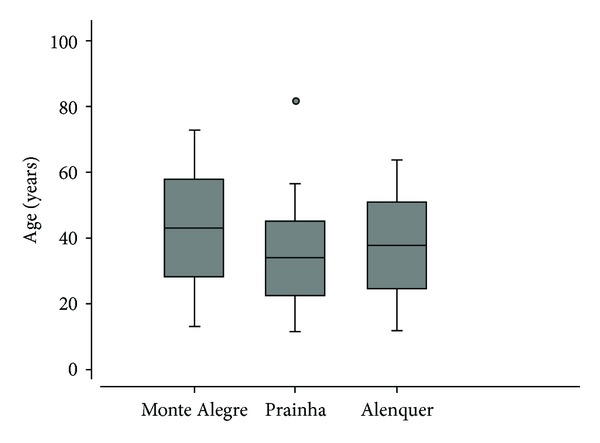
Comparison of age in populations exposed to radiation. Variation of age range with 2 standard deviations comparing the 3 populations according to the ages of the sampled individuals.

Individuals ranged from 17 to 83 years old, with an average age of 39.5 years and a median age of 38 years. In Monte Alegre, ages ranged from 17 to 77 years old, with an average of 43.8 years and a median of 44 years. In Prainha, ages ranged from 19 to 83 years old, with an average of 35.7 years and a median of 31.5 years. Ages in Alenquer ranged from 20 to 74 years old, with an average of 38.6 years and a median of 37 years. The Monte Alegre municipality had a significantly greater number of elderly individuals compared to the other municipalities (Kruskal-Wallis test; *P* < 0.05). This difference was greater for Prainha (*P* = 0.004) than for Alenquer (*P* = 0.093, Figure 3). Additionally, when comparing those above 50 years of age among the different municipalities, Monte Alegre still had a greater number of elderly individuals (*P* < 0.05).

Eight individuals, all female, were diagnosed with cancer: 4 from Monte Alegre, 1 from Prainha, and 3 from Alenquer. We performed a followup of these subjects to determine their survival. The cancer types found by municipality and survival are described in Table 2. The remaining 155 were clinically healthy. Estimates of cancer incidence for the Brazilian population in the period studied were approximately 243.28 cases per 100,000 inhabitants [50]. The number of cancer cases found in the study population is statistically higher than that estimated for Brazil in the same period (*P* < 0.001).

### 3.2. Mutations Identified in Gene *TP53*

A total of 755 sequences (forward and reverse) were generated and analyzed to investigate the nucleotide mutations present in exons 4, 5, 6, 7, and 8 of *TP53* and in the parts of the introns closest to these exons; a total of 191,921 base pairs were analyzed.

**Figure 4:**
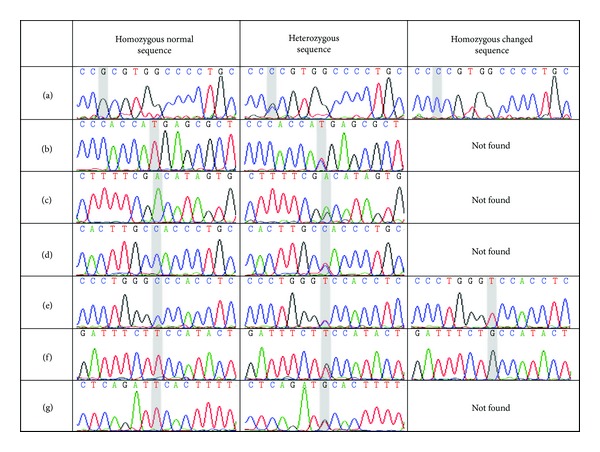
Nucleotide alterations found. (a) Arg72Pro; (b) 1 T → C exchange in third nucleotide of codon 179 (exon 5) that did not change the amino acid histidine; (c) rs800372; (d) rs7880172; (e) rs12947788; (f) rs12951053; (g) 1 T → G exchange in intron 8 at position 13,958.

The following nucleotide mutations were observed in the studied individuals: (1) the Arg72Pro exchange; (2) 1 T → C exchange at the third nucleotide of codon 179 (exon 5), which did not change the amino acid histidine; (3) 1 A → G exchange at the third nucleotide of codon 213 (exon 6), which did not change the amino acid arginine (rs800372); (4) 3 exchanges in intron 7 (C → T at position 13,436 [rs7880172], C → T at position 13,491 [rs12947788], and T → G at position 13,511 [rs12951053]); (5) 1 T → G exchange in intron 8 at position 13,958 (Figure 4). Allele frequencies and observed genotypic changes are described in Table 3.

A comparison of the allele frequencies observed in the study with those of the global population, as described in GenBank [16], is shown in Table 4; there were no differences between the frequencies.

The mutations occurred in all investigated populations, with the exception of rs7880172, which occurred only in Alenquer, and the T → G polymorphism at position 13,958 (intron 8), which was detected only in Monte Alegre and Alenquer.

 Of the 151 samples used for genetic analysis, 148 were screened for PIN3 duplication. The duplication was present in 52 individuals from Monte Alegre, 48 from Prainha, and 48 from Alenquer. Four individuals (1 from Monte Alegre and 3 from Prainha) were homozygous for the duplicated region, 25 were heterozygous, and 119 had no duplication. The allele frequency of PIN3 was 0.111 (33/296).

The linkage disequilibrium analyses demonstrated that the mutations corresponding to PIN3 and Arg72Pro were in strong linkage disequilibrium (*P* < 0.0001), as were rs12947788 and rs12951053 (*P* < 0.0001). There was also linkage disequilibrium between Arg72Pro and polymorphisms rs12947788, and rs12951053 (*P* = 0.0003). Marginally, significant linkage disequilibrium was observed between PIN3 and polymorphisms rs12947788 and rs12951053 (*P* = 0.0482). Other haplotypes showed no linkage disequilibrium.

All mutations found were in Hardy-Weinberg equilibrium (*P* > 0.10), with the exception of rs12947788 and rs12951053 (*P* = 0.008). Differences in the presence of the various mutations among the populations of Monte Alegre, Prainha, and Alenquer were not statistically significant (*P* > 0.10).

The allele frequency of the Arg72Pro polymorphism did not differ significantly between the study populations and the 220 control samples (*P* > 0.1) (Table 5). Arg72Pro was the only polymorphism for which the allelic and genotypic frequencies of the study populations were compared with the control population. The other mutations identified in the study were compared with data obtained from the general population.

The T → C variants in codon 179 (exon 5) and T → G variants at position 13,958 (intron 8) have not been previously described in population studies. Compared to the other mutations, the differences between those observed in this study and those in the global population were not statistically significant (*P* > 0.50). There was no association between these polymorphisms and any factors from the social, clinical, and environmental survey (*P* > 0.05).

### 3.3. Observed Nucleotide Mutations and Genetic Susceptibility

All identified mutations were analyzed for possible associations with age, gender, ethnic origin, smoking, drinking, family history of cancer, personal history of cancer, reproductive difficulty, and reports of miscarriage.

Genotype *TP53* ^Arg72Pro^ *C/C *was effectively associated with individuals who reported having cancer (*P* = 0.024; OR = 6.48; CI: 1.29–32.64) and corrected for old age and smoking (Table 6). The other social, clinical, and environmental factors were not considered, as they were not statistically significant.

## 4. Discussion

Some uranium isotopes have chemotoxic and genotoxic properties due to radiation released in the decay process [7, 8]. Nonmutated p53 normally responds to radiation with a high level of expression and subsequently mediates cell cycle arrest and DNA repair activation [51]. Therefore, this protein is important for monitoring radiosensitivity to both high and low doses of radiation, alone or in combination with other stressors [52].

Age greater than 50 years was expected to be a risk factor for the development of carcinogenesis in Monte Alegre, Prainha, and Alenquer because old age implies a longer exposure to radiation, compounded by the fact that repair mechanisms in elderly people are less efficient [53, 54]. Nevertheless, in this work, advanced age was not significantly associated with the development of cancer in any study region. A plausible explanation may be that the dose of radiation in the reserve is too low to cause significant damage to DNA.

The proportion of cancer cases observed in the study population was greater than that expected in Brazil during this time period. Exposure to uranium is related to an increased risk of developing cancer, particularly leukemia [55–57], the type of cancer most prevalent in this study (3/8).

In this work, a single nucleotide mutation that results in an amino acid change was detected in exon 4 (Arg72Pro). This polymorphism resulted in the exchange of arginine (CGC) for proline (CCC) at codon 72. The protein containing proline is less effective as a transcription factor (which normally interrupts the cell cycle, activates the apoptotic pathway, activates the expression of genes related to DNA repair, and represses oncogenes) and increases the susceptibility to cancer development [32]. Therefore, individuals with genotype *TP53* ^Arg72Pro^ *C/C *are more radiosensitive and are subjects for public health action in regions with environmental radioisotopes.

Although it has been associated with several types of cancer [34–36], the 16-base pair duplication in intron 3 did not show such an association in our study. The duplication does not have a causal relationship with the development of cancer [38], which may explain the lack of association in our study.

Being homozygous for Arg72Pro was correlated with a six-fold increase in the risk of developing cancer (*P* = 0.024; OR = 6.48; CI: 1.29–32.64). This finding corroborates several studies that found an association of the polymorphism with carcinogenesis susceptibility [30, 31, 58, 59]. In contrast, other studies of the polymorphism did not report an association with cancer development [60–62]; this may be because the polymorphism is a susceptibility factor rather than a determining factor in the development of neoplasias. Therefore, individuals with genotype *TP53* ^Arg72Pro^ *C/C *may develop cancer when exposed to multiple factors, including mutagenic and carcinogenic factors such as radiation [26]. Additionally, a multiple logistic regression of the Arg72Pro homozygosity data for the individuals who reported having cancer with social, clinical, environmental, and genetic factors was performed, but no association was identified.

The strong linkage disequilibrium between Arg72Pro and polymorphisms PIN3 (*P* < 0.0001), rs12947788 (*P* = 0.0003), and rs12951053 (*P* = 0.0003) confirms the findings of other studies [63, 64] and demonstrates that the modification serves as a marker of susceptibility and not as a consequence of mutational low doses of ionizing radiation.

Nucleotide mutations that had not previously been described in population studies (T → C at position 179, exon 5, and T → G at position 13,958, intron 8) were observed in these populations.

In populations chronically exposed to genotoxic agents, such as natural radioactivity from the environment, *TP53 *is of great importance because previous exposure to ionizing radiation may provoke loss of or reduction in the efficacy of the maintenance of genomic stability involving the gene [51]. 

The effect of low doses of ionizing radiation depends on a number of factors, including the individual's genetic constitution, the type of tissue affected, and the level of cellular stress [65, 66]. Moore et al. [67] observed *in vitro *that the sensitivity to the genotoxic effects of ionizing radiation depends on the efficiency of *TP53* expression. Our work suggested that individuals with genotype *TP53* ^Arg72Pro^ *C/C *had differences in sensitivity to uranium exposure. Consequently, exposed populations must be monitored with specific public health measures to minimize the possible effects of radioactivity.

A recent study in the same populations reported no increases in the frequency of chromosomal breaks or DNA fragility in individuals living in the area of mineralization [68].

Several genes are involved in carcinogenesis, and many of them are related to the proper functioning of *TP53, *either by regulating or being regulated by this gene. Therefore, the neoplasic process may not be exclusively dependent on the defective gene; rather, changes to the gene may contribute to tumor development [25]. As a result, *TP53 *may be an important biomarker of susceptibility to carcinogenic agents given its importance in tumorigenesis.

## 5. Conclusions

The analysis of the most important regions of gene *TP53 *allowed the detection of polymorphisms Arg72Pro, PIN3, rs800372, rs7880172, rs12947788, and rs12951053. Nucleotide alterations not yet described in population studies (T → C in third nucleotide of codon 179, exon 5; T → G at position 13,958, intron 8) were also observed.

Frequencies of the alterations in the studied populations and the global population were not statistically different. However, a homozygous polymorphism in Arg72Pro was an important risk factor for cancer development (*P* = 0.024; OR = 8.48; CI: 1.41–51.64), when adjusted for age and smoking. This association may be because the context of chronic exposure to low dose of radioactivity. However, further studies are needed that measure the amount of radioactivity absorbed by individuals to demonstrate this association.

## Figures and Tables

**Table 1:** Target regions, primer sequences, and size of amplified fragments.

Region	Target	Primer sequences	Product size (bp)
PIN3	Intron 3	5′ GGGACTGACTTTCTGCTCTTGT 3′	147 or 163
		5′ GGGACTGTAGATGGGTGAAAAG 3′	
Region 1	Exon 4	5′ TTGCCGTCCCAAGCAATGGATGAT 3′	199
		5′ TCTGGGAAGGGACAGAAGATGAC 3′	
Region 2	Exon 5 and 6	5′ GCCGTCTTCCAGTTGCTTTA 3′	488
		5′ TAACCCCTCCTCCCAGAGAC 3′	
Region 3	Exon 7	5′ TTGGGCCTGTGTTATCTCCT 3′	253
		5′ TGATGAGAGGTGGATGGGTAG 3′	
Region 4	Exon 8	5′ CAAGGGTGGTTGGGAGTAGA 3′	331
		5′ TGCTAGGAAAGAGGCAAGGA 3′	

**Table 2:** Description of cancer types found by municipality and survival.

Population	Subject	Age (years)	Cancer type	Survival
Monte Alegre	1	46	Acute myeloid leukemia	42 months
	2	26	Lung cancer	48 months
	3	55	Chronic lymphocytic leukemia	49 months
	4	24	Osteosarcoma	29 months
Prainha	5	19	Chronic lymphocytic leukemia	38 months
Alenquer	6	54	Gastric cancer	48 months
	7	51	Osteosarcoma	35 months
	8	44	Hepatocarcinoma	28 months

**Table 3:** Allele and genotype median frequencies observed *TP53* gene from investigation populations.

Frequencies	Arg72Pro (exon 4)	His179His (exon 5)	rs800372 (exon 6)	rs7880172 (intron 7)	rs12947788 (intron 7)	rs12951053 (intron 7)	13,958 (intron 8)
	GG 0.457	TT 0.980	AA 0.940	CC 0.986	CC 0.788	TT 0.788	TT 0.980
Genotype	GC 0.424	TC 0.020	AG 0.060	CT 0.014	CT 0.172	TG 0.172	TG 0.020
	CC 0.119	CC 0.000	GG 0.000	TT 0.000	TT 0.040	GG 0.040	GG 0.000
Allele	G 0.669	T 0.990	A 0.970	C 0.993	C 0.874	T 0.874	T 0.990
	C 0.331	C 0.010	G 0.030	T 0.007	T 0.126	G 0.126	G 0.010

**Table 4:** Comparison between the allelic frequencies found in the study and described for the global population.

Alterations	Allelic frequency		
	Study	Global [16]	*P*
Arg72Pro (exon 4)	0.331	**0.352**	**>0.05**
His179His (exon 5)	0.010	**Not described**	**Not applied**
rs800372 (exon 6)	0.030	**0.011**	**>0.05**
rs7880172 (intron 7)	0.007	**0.006**	**>0.05**
rs12947788 (intron 7)	0.126	0.159	**>0.05**
rs12951053 (intron 7)	0.126	**0.157**	**>0.05**
13,958 (intron 8)	0.010	**Not described**	**Not applied**

**Table 5:** Allele and genotype frequency observed in Arg72Pro from investigated and control populations.

	Populations				
Frequencies	Belém*	Monte Alegre	Prainha	Alenquer	
	GG 0.482	GG 0.404	GG 0.520	GG 0.449	
Genotype	GC 0.409	GC 0.481	GC 0.380	GC 0.408	
	CC 0.109	CC 0.115	CC 0.100	CC 0.143	
Allele	G 0.686	G 0.644	G 0.710	G 0.653	*P* > 0.1
	C 0.314	C 0.356	C 0.290	C 0.347	

*Control population.

**Table 6:** Analysis by multiple logistic regression of the cancer association with risk factors.

Variable	Value of *P*	Odds ratio	Confidence interval (95%)
*TP*53^*Arg*72*Pro*^ *C/C *	0.024	6.48	1.29–32.64
Age (over 50)	0.098	3.92	0.78–19.80
Smoking	0.296	0.40	0.07–2.22
